# Applicability of Existing Gender Scores for German Clinical Research Data: Scoping Review and Data Mapping

**DOI:** 10.2196/74162

**Published:** 2026-01-08

**Authors:** Lea Schindler, Hilke Beelich, Elpiniki Katsari, Daniele Liprandi, Sylvia Stracke, Dagmar Waltemath

**Affiliations:** 1 Medical Informatics Laboratory, University Medicine Greifswald, Walther-Rathenau-Straße 48, Greifswald, 17475, Germany, 49 03834 86 ext 8370; 2 Heart Surgery, University Medicine Greifswald, Greifswald, Germany; 3 Laboratory of Evolutionary Biomechanics, Zoological Institute and Museum, University Greifswald, Greifswald, Germany; 4 Internal Medicine A, Nephrology, University Medicine Greifswald, Greifswald, Germany; 5 Core Unit Data Integration Center, University Medicine Greifswald, Greifswald, Germany

**Keywords:** gender score, gender research, review, clinical routine, clinical research data, German Medical Informatics Initiative

## Abstract

**Background:**

Considering sex and gender improves research quality, innovation, and social equity, while ignoring them leads to inaccuracies and inefficiency in study results. Despite increasing attention on sex- and gender-sensitive medicine, challenges remain with accurately representing gender due to its dynamic and context-specific nature.

**Objective:**

This work aims to contribute to the implementation of a standard for collecting and assessing gender-specific data in German university hospitals and associated research facilities.

**Methods:**

We carried out a review to identify and categorize state-of-the-art gender scores. We systematically assessed 22 publications regarding the applicability and practicability of their proposed gender scores. Specifically, we evaluated the use of these gender scores on German research data from routine clinical practice, using the Medical Informatics Initiative core dataset (MII CDS).

**Results:**

Different methods for assessing gender have been proposed, but no standardized and validated gender score is available for health research. Most gender scores target epidemiological or public health research where questions about social aspects and life habits are already part of the questionnaires. However, it is challenging to apply concepts for gender scoring on clinical data. The MII CDS, for example, lacks all variables currently being recorded in gender scores. Although some of the required variables are indeed present in routine clinical data, they need to become part of the MII CDS.

**Conclusions:**

To enable gender-specific retrospective analysis of routine clinical data, we recommend updating and expanding the MII CDS by including more gender-relevant information. For this purpose, we provide concrete action steps on how gender-related variables can be captured in routine clinical practice and represented in a machine-readable way.

## Introduction

### Background

Taking sex and gender (see Textbox 1 and Textbox 2 ) into account improves the quality of research and care, and it supports social equity [1]. Considering sex and gender from the outset can lead to new discovery and foster innovation. Ignoring sex and gender, however, leads to inaccuracies, inefficiencies, and difficulties generalizing results. This is, for example, highlighted by a recent study showing that men with low femininity reported a significant decrease in anxiety during the COVID-19 pandemic, meanwhile women with low femininity reported a significant increase [2].

Textbox 1. **Definition of biological sex [3,4].**

Biological sex is defined through biological attributes, such as:

- Genetics (ie, chromosomes, gene expression)
- Hormone levels
- Anatomy (ie, internal and external sex organs/reproductive organs)

Deviations regarding the hormonal or chromosomal attributes are possible, where the external appearance can be intersex and it is not possible to decide.

Textbox 2. **Definition of (social) gender [3,4].**

Gender refers to socially constructed roles, behaviors, expectations, and norms associated with being a woman, man, girl, or boy. It is formed through biological and psychosexual factors and an individual’s social biography, and it is shaped by one’s role in society. Gender influences how people perceive themselves and others and how they act and interact.

Many health care decisions are influenced by the patients’ sex and gender, through social norms and experiences. Sex- and gender-sensitive medicine is receiving increasing attention in international research [5]. However, several publications have shown that women are still not considered equally to men and are poorly represented due to biased study designs [6-8]. It is well known today that this practice leads to an increase in inequality in medical treatment due to inappropriate therapies. Moreover, sex- and gender-specific reporting is often deficient, which leads to a lack of reproducibility and reduced effectiveness of research studies [1].

Even though the situation has improved, it can still be challenging to represent psychosocial gender aspects, as gender is a context-specific construct that is dynamic and multidimensional and can differ across time, geographical regions, and societies [9].

Gender scores are developed and used to assess gender roles and gender identity. They can be created and applied retrospectively to extract frequently missing gender information from already existing data. For this purpose, gender-sensitive variables in the existing data are determined by expert knowledge or statistical algorithms. Gender scores can then be calculated to predict social gender roles. For example, Lacasse et al [10] and Nauman et al [11] each developed a retrospective gender score and applied it to specific datasets from population studies. Examples for the corresponding gender-sensitive variables are specific *professions*, like in health care or construction, and typical *personality trait*s, like agreeableness or neuroticism; *loneliness* or *stress* was considered. In general, retrospective scores cannot capture participants’ current gender identity (see Textbox 3).

Textbox 3. **Definition of gender identity [3,4].**

Gender identity is determined through individual self-perception and the sexual identity of a person, which may differ from someone’s physical appearance or biological sex.

Prospective assessments can enhance the collection of gender-specific data from the outset of a study by incorporating further social data or information regarding the identity. Fraser et al [12] and Lagos and Compton [13], for example, evaluated a prospective 2-step gender identity measure on data from huge studies. The 2-step measure differentiates between a person’s current gender identity and birth-assigned sex (see Textbox 3 and Textbox 4). The given examples show that gender scores are mostly used in surveys and studies and hardly used on routine clinical data.

Textbox 4. **Definition of birth-assigned sex (BAS) [3,4].**

BAS refers to the information that can be found on official documents, like a birth certificate, and is assigned based on the child’s external anatomy or sex organs. BAS is often measured by a binary choice of response (ie, male or female). Biological sex is not a dichotomy, nor is BAS, where diversity is given.

All 38 university hospitals in Germany are part of the Medical Informatics Initiative [14] (MII), which serves to close the gap between research and routine care. A core dataset (CDS) is being developed by the MII consortia and the participating university hospitals to foster interoperability and allow a shared use of routine clinical data across Germany. Data Integration Centers collect and process routine clinical data and make them accessible to researchers for secondary use. If and how this established dataset considers gender and gender score applications are open questions.

It is important to note that the discussion about the applicability and state of gender scores does not only affect Germany. Critical assessments of sex and gender scores reveal that the problem affects different western cultures [15-18]. Ignoring gender differences in clinical research is a widespread global issue that undermines the validity and applicability of scientific findings across diverse populations [16]. A study conducted in Australia using the Bem Sex Role Inventory (BSRI) [19] found that the majority of participants received different gender scores in at least 1 of the 3 consecutive years, highlighting the complexity and fluid nature of gender identity [18]. This is particularly concerning because the BSRI yielded inconsistent results even among a relatively stable population of patients older than 75 years — where minimal shifts in habits or identity would be expected over time. Despite being based on outdated social stereotypes, the BSRI is one of the most used gender scores, and other scores are partly based on it, as we later describe.

In this context, where classic gender scores are struggling, new tools are being considered to annotate medical data according to the gender of patients, thanks to the rise of machine learning methods [20]. The results of such methods can then be systematically used to mitigate biases in research, as done by Neufang et al [21], who developed an artificial intelligence model to improve the fairness of attention deficit hyperactivity disorder diagnosis with respect to gender. It is important to understand whether these algorithms are also applicable to general clinical data datasets, such as the MII CDS, to allow large-scale integration of gender debiasing in future research.

The issues outlined in the previous paragraphs indicate that more work needs to be done to update gender scores, taking into account the changes in commonly defined social rules that have occurred in the last century. In practice, outdated scores, such as the BSRI, are often used without considering proper validation and potential biases [22]. Moreover, a gold standard is still missing for how sex and gender should be considered in research with clinical research data. A practicable score should be balanced, comprehensive, and easily applicable. Interestingly, no gender score has so far been established as an international standard.

We therefore conducted a systematic evaluation of gender scores regarding their applicability in health research using German efforts on Data Integration in Medicine as a benchmark. As the world’s third-largest economy and one of the World Health Organization’s top two donors, we expected the German MII CDS to be a good candidate for our evaluation. In this article, the term gender score refers to the assessment of gender in general (social gender and gender identity), and the term gender identity measure refers exclusively to gender identity.

### Related Work

The focus of our scoping review protocol published in JMIR Review Protocols [23] was the applicability and practicability of gender scores in health research. Related to the work described in this paper, Horstmann et al [24] published a review on recently used instruments for the operationalization of sex and gender in health research, and the work by Miani et al [25] summarizes epidemiological aspects in gender scores. The reviews included publications until 2020 and 2021, respectively. For this work, we identified the latest scores and therefore deliberately limited our search to publications between 2019 and 2024. Of the 18 articles from our primary result set, 13 were published after 2020.

In parallel to our work on the systematic review of recent gender scores, another systematic review about the operationalization of gender via composite gender scores in epidemiological studies was published in 2024 by Ballering et al [26]. The authors searched 3 databases (PubMed, Web of Science, and CINAHL) and identified 24 articles with a total of 26 gender scores developed in Europe or North America. They aggregated information on gender scores regarding author, year of publication, methodology, cohort information, and the included variables of the respective gender score and found that many variables overlapped across multiple studies.

Ballering et al [26] criticized theory-driven approaches — developed solely based on expert knowledge — as experts are not free from bias, potentially enhancing sexism or other biases. The authors further claim that data-driven approaches — using statistical models to develop gender scores — only predict female or male sex through identified psychosocial variables and do not consider gender itself. Psychosocial variables that do not associate with sex are excluded, and the quality of the gender scores is strongly dependent on the quality of the dataset. Overall, Ballering et al [26] concluded that data-driven approaches might be good to personalize health care, as gendered variables point out differences between women and men that could lead to improved treatment decisions and patient care.

The work by Ballering et al [26] is a great foundation for our work, and we are able to contribute further insights on the applicability of gender scores on routine clinical data. We had an overlap of 13 articles on both sides, with 13 of 22 articles in our review and 13 of 24 articles in the review by Ballering et al [26] (ie, there was an overlap of more than 50%: 59% ours and 54% theirs). We found further articles regarding gender identity that can be applied from the outset of a study or data collection, which could be of great use to implement gender information in routine clinical practice.

### Aim of This Work

In the scope of this work, we identified and categorized state-of-the-art gender scores, systematically assessed their applicability and practicability, and evaluated their applicability on German research data from routine clinical practice (see the Applicability of Gender Scores on the MII CDS section).

**Figure 1.**
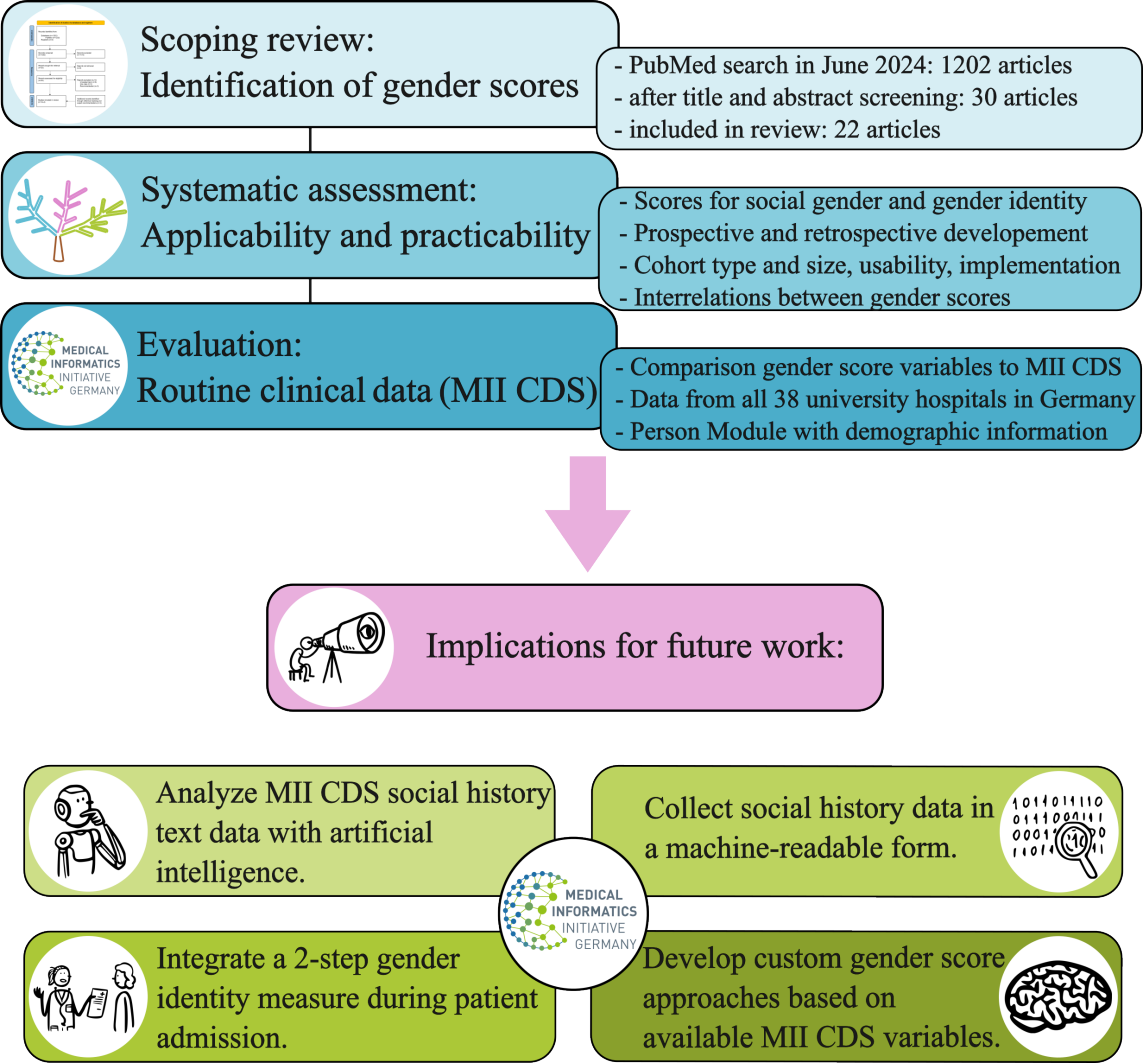
Overview of the implemented methods. MII CDS: Medical Informatics Initiative core dataset.

The process of our work is illustrated in Figure 1. This work aimed to contribute to the necessary implementation of a standard for collecting and assessing gender-specific data in German university hospitals and the respective research facilities; therefore, we formulated 4 action steps for an extension in health research to enable gender-specific analyses (see Figure 1), which are shown at the bottom of the figure and will be described in detail in the Discussion section.

## Methods

### Scoping Review

We conducted a scoping review to identify articles that reported on the development and implementation of gender scores in health-related studies (see Figure 1). It resulted in 22 articles, which were identified through a title and abstract screening followed by a full-text evaluation. Moreover, we developed a data charting form to extract information on article type and applicability of the gender score in health research. The review protocol was published in JMIR Research Protocols [23].

### Assessment of Applicability and Practicability

To assess the applicability and practicability of gender scores, we followed a systematic approach (see Figure 1). In the scope of this work, we extended the data charting process to fully assess the applicability of gender scores on routine clinical data.

#### Social Gender and Gender Identity

First, we distinguished between scores for gender identity (see Table 1) versus social gender (see Tables2  3). Gender identity (see Textbox 3) is usually measured in a prospective way because it is a purely subjective perception and it is not possible to extract this information when it was not collected. However, social gender includes norms that are expected in society, stereotypes, and certain behaviors (see Textbox 2), and it can be measured prospectively and retrospectively. Typical behavior (eg, specific hobbies, occupational segregation, doing housework or odd jobs) can be analyzed and interpreted after data collection. A typical female or male lifestyle or behavior will lead to different exposure to risk factors for accidents or disease. Social expectations will also influence the process of recovery.

**Table 1.** Prospective gender identity measures [12,13,27,28].

Author(s) (year)	Description	Questions and answer option
Lagos and Compton (2021) [13]	2-step gender identity measure	What sex were you assigned at birth? (male, female, intersex) What is your current gender? (woman, man, transgender, a gender not listed here [space for answer])
Fraser et al (2020) [12]	Open-ended gender measure	What is your gender? (open-ended item)
McGuire et al (2019) [27]	Genderqueer Identity Scale	I am non binary, genderqueer, or an identity other than male or female. I don’t want to be seen in the gender binary (as either male or female) I try to deliberately confuse people about whether I am male or female. I try to do things that are masculine and feminine at the same time. I enjoy it when people are not sure if I am male or female The way I think about my gender has always been the same. My gender comes naturally from within me. My gender is something I have spent a lot of time figuring out. The way I show my gender changes depending on who I am with. The way I think about my gender has been influenced by experiences in my life. The way I think about my gender will probably continue to change further as I age I have done research about gender theory and gender roles. I try to convince others that society should not insist on a gender binary. I try to convince others that society expects people to be too gender conforming. Around me, I make sure people are free to express whatever gender roles they want. The way I show my gender is important because I push society to question traditional gender roles. I encourage others to be more open minded about gender and gender roles. In the future, my gender expression will be traditional. In the future, it will upset me if people misgender me. The way I show my gender will probably be mostly the same from day to day. In the future, I expect that people will rarely question my gender. In the future, I think my gender will be fluid or change over time. I will have a non-traditional gender role (be gender non-conforming).
Lombardi and Banik (2016) [28]	2-step gender identity measure	What is your sex or gender? (Check ALL that apply; male, female, other: please specify [space for answer]) What sex were you assigned at birth? (Check one; male, female, unknown or question not asked, decline to state)

**Table 2.** Prospective gender scores regarding social gender [17,19,20,29-32].

Author(s) (year)		Gender score variables
Pohrt et al (2022)^a^ [29]		Level of education^b^ Civil status^b^ Personal net income Households primary earner status Responsibility for caring for other people^b^ Responsibility educating and upbringing people^b^ Responsibility for picking up people when they become ill Hours per week spent on housework Being mainly responsible for housework, Social support^b^ PAQ^c^ BSRI^d^: masculinity score BSRI: femininity score Perceived social standing
Tibubos et al (2022) [30]		
PAQ-8		Not at all emotional - very emotional Not at all aware of feelings of others -very aware of feelings of others Never gives up easily - gives up very easily Not at all self-confident - very self-confident Feels superior - feels very inferior Not at all understanding of others - very understanding of others Very warm in relations with others - very cold in relations with others Goes to pieces under pressure - stands up well under pressure
Demuth et al (2021) [31]		The questionnaire is not available.
Nielsen et al (2021) [17]		Caregiver strain^b^ Work strain^b^ Independence Risk-taking Emotional intelligence Social support^b^ Discrimination
Pelletier et al (2015) [20]		Primary earner status Personal income Number of hours per week doing housework Primary responsibility for doing housework Level of stress at home^b^ BSRI: masculinity score BSRI: femininity score
Spence et al (1975) [32]		
PAQ: male score		Independent Not easily influenced Good at sports Not excitable, minor crisis Active Competitive Skilled in business Knows ways of world Adventurous Outspoken Interested in sex Makes decisions easily Not give up easily Outgoing Acts as leader Intellectual Self confident Feels superior Takes a stand Ambitious Stands up under pressure Forward Not timid
PAQ: female score		Emotional Not hide emotions Considerate Grateful Devotes self to others Tactful Strong conscience Gentle Helpful to others Kind Aware, other feelings Neat Creative Understanding Warm to others Likes children Enjoys art and music Expresses tender feelings
PAQ: sex-specific score		Aggressive (M) Dominant (M) Likes math and science (M) Excitable, major crisis (F) Home-oriented (F) Mechanical aptitude (M) Needs approval (F) Feelings hurt (F) Cries easily (F) Loud (M) Religious (F) Sees self running show (M) Needs for security (F)
Bem (1974) [19]		
BSRI: masculinity score		Acts as a leader Aggressive Ambitious Analytical Assertive Athletic Competitive Defends own beliefs Dominant Forceful Has leadership abilities Independent Individualistic Makes decisions easily Masculine Self-reliant Self-sufficient Strong personality Willing to take a stand Willing to take risks
BRSI: femininity score		Affectionate Cheerful Childlike Compassionate Does not use harsh language Eager to soothe hurt feelings Feminine Flatterable Gentle Gullible Loves children Loyal Sensitive to the needs of others Shy Soft spoken Sympathetic Tender Understanding Warm Yielding
BRSI: social desirability score (neutral)		Adaptable Conceited Conscientious Conventional Friendly Happy Helpful Inefficient Jealous Likable Moody Reliable Secretive Sincere Solemn Tactful Theatrical Truthful Unpredictable Unsystematic

a Overlaps with the already published review by Ballering et al [26].

b Variables that are potentially covered in routine clinical care in Germany.

c PAQ: Personal Attributes Questionnaire.

d BSRI: Bem Sex Role Inventory.

**Table 3.** Retrospective gender scores regarding social gender [9-11,33-40].

Author(s) (year)		Gender score variables
Cipriani et al (2024)^a^ [36]		Hostile behavior during childhood Hostile behavior during adulthood Having at least a secondary school diploma^b^ Having at least a bachelor’s degree^b^ Sleep satisfaction^b^ Sleep efficacy^b^ Having private housing Sexual violence during childhood
Gisinger et al (2023)^a^ [35]		Level of education^b^ Household income Household size^b^ Civil status^b^ Perceived life stress Sense of belonging to community
Teterina et al (2023)^a^ [37]		
ICD-10-CA^c^ diagnostic codes with highest effects for predicting male/female gender: male score		Fall on and from scaffolding^b^ Occupant of heavy transport vehicle injured in noncollision transport accident, driver, traffic accident^b^ Driver of other all-terrain or other off road motor vehicle injured in traffic accident^b^ Motorcycle rider injured in collision with fixed or stationary object, driver, traffic accident^b^ Asymptomatic human immunodeficiency virus (HIV) infection status^b^ Motorcycle rider injured in collision with car, pick-up truck or van, driver, traffic accident^b^ Motorcycle rider injured in noncollision transport accident, driver, nontraffic accident^b^ Motorcycle rider injured in unspecified nontraffic accident^b^ Fracture of malar and maxillary bones, LeFort 2, closed^b^ Contact with nonpowered hand tool^b^
ICD-10-CA diagnostic codes with highest effects for predicting male/female gender: female score		Assault by spouse or partner Esthetic sports^b^ Physical abuse^b^ Horse riding sports^b^ Animal-rider or occupant of animal-drawn vehicle injured by fall from or being thrown from animal or animal-drawn vehicle in noncollision accident^b^ Fall while being carried or supported by other persons^b^ Other specified gymnastic and esthetic sports and recreational activity^b^ Bitten or struck by dog^b^ Animal-rider or occupant of animal-drawn vehicle injured in other and unspecified transport accidents^b^ Problems in relationship with spouse or partner
Vader et al (2023)^a^ [38]		
Masculine gender score		Work and education (compared with partner)^b^ Informal care: household chores, odd jobs, taking care of sick people^b^ Lifestyle: physical intensity, type of sport, smoking, alcohol^b^ Emotions: emotional problems, nervousness, energetic and vibrant, exhausted and tired
de Breij et al (2022)^a^ [33]		Working hours^b^ Income Occupation segregation^b^ Education^b^ Informal caregiving^b^ Time spent on household chores
Wandschneider et al (2022)^a^ [9]		A person who is living with their partner for the long term should get married. Children below the age of 6 suffer if their mother works. A same-sex couple can raise a child just as well as a man and woman. It would be good for society if transgender people were recognized as normal. Working experience part-time employment Hours/weekday housework Hours/weekday repairs Hours/weekday leisure, hobbies Worried about global terrorism Worried about crime in Germany Satisfaction with housework Willingness to take risks Worried about own retirement pension
Nauman et al (2021)^a^ [11]		Chronic stress Civil status^b^ Risk-taking behavior Agreeableness Neuroticism Extraversion Conscientiousness Loneliness Level of education
Yuan et al (2021)^a^ [39]		Risk willingness Loneliness^b^ Less participation in household tasks Regular drinking^b^ Depression^b^
Ballering et al (2020)^a^ [40]		Leisure activities (eg, type of sport)^b^ Occupation-related components (eg, profession, housewife/-husband)^b^ Time spend on household tasks Time spend on odd jobs Lifestyle (eg, dieting, preparing dinner, alcohol, smoking) Experiencing long-term difficulties or negative life events Personality traits and emotions (eg, discipline, impulsiveness, self-consciousness, vulnerability, competence
Lacasse et al (2020)^a^ [10]		Civil status^b^ Racial/ cultural group Level of education^b^ Household income, size, and composition^b^ Household food insecurity Ownership of the household Sense of belonging to the local community Occupation: type, working hours, self-employment^b^ Amount of stress
Smith and Koehoorn (2016)^a^ [34]		Responsibility for caring for children^b^ Occupational segregation^b^ Hours of work relative to partner/spouse Education relative to partner/spouse^b^

a Overlaps with the already published review by Ballering et al [26].

b Variables that are potentially covered in routine clinical care in Germany.

c ICD-10-CA: International Statistical Classification of Diseases and Related Health Problems, Tenth Revision, Canada.

#### Prospective and Retrospective Scores

Second, we distinguished between prospective (see Tables1  2) and retrospective (see Table 3) scores. It is advisable to include gender variables from the outset of a study; this results in a prospective gender score and is easy to apply to the study dataset. However, the huge amount of existing data drives the development of retrospective gender scores, where gender-specific information can be extracted in hindsight.

#### Cohort Type and Size

Third, it needs to be considered whether the gender scores themselves are representative. In practice, some gender scores are developed on restricted datasets, for instance, for specific patient groups or other samples that are not representative (eg, older age groups, workers, or specific diagnoses). This is underlined by the finding of Wandschneider et al [9] who showed that gendered practices vary between eastern and western Germany, reflecting a different social development due to the historical political context. As gender norms, stereotypes, or behaviors vary between societies, the relevant gender variables can differ across social groups, countries, and regions or even between different societies within a country.

Furthermore, since retrospective scores depend on statistical modeling, we also considered the sample size on which the models were based. Another analysis we performed was to further classify the background under which the score was developed. We took into account whether the research group used patient or population data to categorize which gender score might be more appropriate for the MII CDS. We also considered the continent where the data collection took place, which is important for consistency due to varying gender-sensitive variables depending on the society or geographical location.

#### Usability

Fourth, we examined the validity, usability, and practicability of the gender scores. Validity is the main issue because it is of high importance that the score is representative regarding gender. Usability refers to how well the score is established. If a new standard is to be introduced, it is of high importance to use an accepted model to ensure that the scores are usable, comparable, and applicable to large-scale studies and assessments. Practicability refers to how difficult a score would be to integrate into health research.

#### Implementation

The fifth point we included in our assessment was the level of implementation of scores. To apply scores systematically on existing data, a theoretical model was not sufficient. Instead, it was necessary to have the executable code of a model to integrate it directly. Therefore, we further investigated the applicability of gender scores from the technical viewpoint to evaluate whether models can, realistically, be implemented in a clinical setting. We used the following categories to describe this aspect: code published — refers to a gender score for which an executable model was published; data published — refers to a gender score for which the underlying data are available and a score could be reproduced; statistical parameters published — refers to a model for which all statistical parameters were published and the model could be constructed from them; not published — a model for which not all relevant parameters are available. We considered “code published” the gold standard regarding applicability. “Data published” and “parameters published,” on the other hand, might contain sufficient information to construct an executable model. In the scope of this study, we considered code or data available “on request” as unavailable. However, in practice, there might still be limitations if the description of the article is not concise enough to reproduce all steps.

#### Interrelations

**Figure 2.**
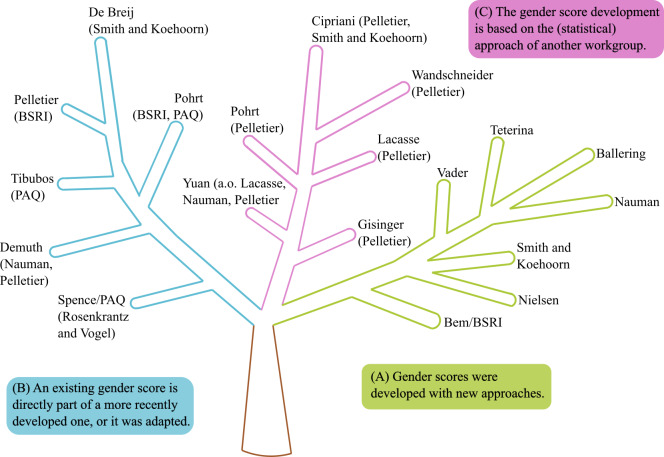
Interrelations between gender scores, where the first 1 or 2 author names identify the work and the developed gender score (see Tables2  3) [9-11,17,19,20,29-41]: (A) gender scores developed with completely new approaches and methods are shown in green; (B) the included or adapted gender scores are in parentheses in the blue branch; (C) gender scores developed based on previous methods and statistical approaches, which are shown in parentheses, are shown in pink. BSRI: Bem Sex Role Inventory; PAQ: Personal Attributes Questionnaire.

Based on the extracted data, we performed further analysis to better understand the currently available gender scores. We noticed early on that direct dependencies exist between gender scores. Therefore, we extracted and modeled different types of dependencies between gender scores (see Figure 2). This analysis allowed us to investigate how scores were developed and whether improvements were made to existing gender scores.

One relation between gender scores captures cases in which an existing gender score was used directly or adapted in another score (see Figure 2B). For instance, the gender score from Pelletier et al [20] includes the BSRI [19]. de Breij et al [33] adapted the score from Smith and Koehoorn [34] but changed “caring for children” to “informal caregiving” as the data were available in their dataset (see Figure 2B).

Moreover, development relationships (see Figure 2C) reflect when one gender score was developed based on the approach, for instance the statistical model, of another research group. For example, Gisinger et al [35], with their statistical model of principal component analysis and logistic regression analysis, were inspired by the approach of Pelletier et al [20].

### Applicability on Clinical Routine Data

To assess whether gender scores are applicable on existing data and whether they can be integrated in clinical routine data, we used the technical specification of the MII Core Data Set - Module “Person” as a representative sample against which to evaluate the gender scores (see Figure 1). The MII CDS [14] (see the Background section) is being collected across all 38 university hospitals in Germany. It contains interoperable, consolidated data items with a high degree of standardization in line with international standards to make collaborative data analysis practicable and efficient.

The CDS [42] consists of basic and extension modules that are subject to iterative refinement. The basic modules are generic, and the extension modules are relevant for specific medical fields and disciplines such as oncology or intensive care. We used the *Person Module*, one of the early basic modules, which contains demographic information such as name, date of birth, address, and administrative gender as stated in official documents. The CDS has a comprehensive description of how the data are structured and how the data can be accessed, which allows us to directly compare it against gender scores. The information is available and can be compared based on a sample dataset [43]. Overall, this makes the CDS a suitable choice for studying how gender scores can be applied in realistic clinical data collection.

Retrospective scores required that we compared the variables contained in the gender scores against the variables available in routine clinical data. To perform this comparison, we systematically analyzed all variables contained in the sample dataset of the MII CDS against the variables included in the statistical models of the gender scores.

### Ethical Considerations

We did not collect any personal data, so ethical approval was not needed.

## Results

The full results of the review are presented in Multimedia Appendix 1 to maintain brevity in the main text.

### Scoping Review

Our review results are categorized into prospective development (Tables1^2^) and retrospective development (Table 3) and between gender scores (Tables2^3^) and gender identity measures (Table 1). Articles that overlapped with the already published review by Ballering et al [26] are marked in the tables.

### Assessment of Applicability and Practicability

#### Social Gender, Gender Identity, and Prospective and Retrospective Scores

We distinguished between articles targeting gender identity and articles targeting social gender. We further classified them into prospective and retrospective development and application.

The 4 identified gender identity measures (see Table 1) are prospectively applicable and could be included in future studies for further data collection regarding gender identity. Participants need to be asked explicitly which gender they belong to; this information cannot be analyzed from existing data.

Moreover, we found 18 gender scores to measure social gender in different ways. Of these, 7 are prospective gender scores (see Table 2) that could provide guidance on how to apply (social) gender to a study from the outset. Of the 18 scores, 11 are retrospective (see Table 3) and could be applied on already existing data.

#### Cohort Type and Size

The background of the cohorts is of importance for the practicability. A score contains different variables, depending on whether it was developed on data from population studies or on patient data. The majority of datasets are based on population studies and thus on specific information that is typically collected in these studies. For a complete comparison of cohorts, see our results in Multimedia Appendix 1.

Only 2 gender scores were developed exclusively based on patient data. Teterina et al [37] focused on patients who entered the emergency department with traumatic brain injuries and published an innovative approach to distinguishing gender by diagnostic codes. These diagnostic codes also describe the background of the accident or incident, enabling conclusions to be drawn about gender. Cipriani et al [36] focused on the effects of gender on emergency patients with psychological crisis. Therefore, they used variables assessing childhood trauma, education, sleep quality, and living situation.

Moreover, the article by McGuire et al [27] assessed gender identity during transition through the collection of patient and population data.

However, all of these studies were restricted by the specific patient cohort group and focused on specific conditions. This hampers their applicability on the general public and their usefulness as broadly applied gender scores for routine clinical data.

In addition, the used population cohorts were neither comprehensive nor inclusive. Many studies used a cohort with a specific age group [11,20,29,31,38,39] or with a specific background like being retired [29] or being a worker [10,33]. This is a major weakness for generalizing results, as a gender score based on a limited cohort is not representative of society as a whole and therefore lacks comprehensiveness.

Furthermore, the majority of cohorts used for developing gender scores are based in the same geographic region, primarily Europe and North America. Only one gender identity measure was used on a cohort in Oceania [12]. We could not find a single gender score publication with a cohort from Asia, South America, or Africa. As a result, the developed scores are not representative for every cohort group and are not universally applicable.

With respect to the size of the cohorts, which forms the basis for the statistical development of retrospective gender scores and influences the reliability of statistical models, we noted that some works used huge cohorts or datasets with n>100,000 [35,37,40] and the outstanding cohort size of approximately 700,000 individuals in the study by Smith and Koehoorn [34]. Other gender scores worked with smaller cohort sizes of n<1000 [19,32,33].

#### Usability

We examined the validity of scores, meaning how representative the work is regarding gender. Gender can be different in each society, so it is a major task to implement a gold standard. Some research groups used sex as the dependent variable and, therefore, gender as a predictor for sex (eg, [9-11,20]). This approach can be problematic since gender should be considered beyond biological sex.

Statistical regression approaches can be systematically validated by using a control set to test the generalization of the regression model (eg, by systematic evaluation on a dedicated validation set [20,36,37,40]). Researchers who developed a number of scores using the approach by Pelletier et al [20] did not specify whether they performed a systematic validation of their developed scores [9,29,35]. Nauman et al [11] did not use a validation, and Yuan et al [39] reported a performance value without describing the validation setting they used.

31 20 29 22

Exclusively theory-driven gender scores, where variable scaling is determined by human experts, were not evaluated since there is no gold standard for gender [33,34,38]. Looking at usability, referring to how well established a score already is, some authors did not mention any practical application of their scores [31], whereas other scores were often cited, adapted, and reapplied [19,20,32,34]. Although highly cited scores seem to be widely accepted and used, they might still not be well suited to represent gender. The most cited score was the BSRI, which is also part of other gender scores (eg, [20,29]; see Figure 2), and was often criticized and considered outdated [22].

Regarding practicability, we considered the scope and complexity of questionnaires and how universally applicable variables are on existing data. Some variables were very specific for the used cohort, whereas others frequently appeared in similar studies. Most gender identity measures are well-suited for clinical data collection, being concise and easy to understand, except for the score from McGuire et al [27], which involves 23 questions and focuses on gender transition, making it less suitable. BSRI [19] and PAQ [32] are lengthy prospective gender scores, same as other gender scores containing them. Tibubos et al [30] developed a shorter form of the PAQ with only 8 variables. We also listed different forms of data collection, from self-reporting questionnaires (eg, [20,34]) to interviews (eg, [13,33,42]). The length of forms could be especially relevant if participants complete a self-reporting questionnaire. Interviews tend to be more time-consuming for the staff conducting them.

Regarding retrospective scores, the length does not have such a big impact, but some scores are much shorter than the majority [34,39]. A minimalistic score can be a trade-off between prediction quality and the number of necessary variables. This was, for instance, investigated by Ballering et al [40], who compared the performance between gender scores including 9 and 85 predictive variables.

#### Implementation

Only 3 research groups made their analysis code publicly available (*code published*) and provided reproducible gender scores [9,17,40].

Two studies published their data [17] or used publicly available data [39] (*data published*), which are necessary to evaluate their conclusion. All other works did not publish data, which might be justified by data protection regulations. However, anonymized data could still be helpful for recreating a study.

Statistical parameters were published in most retrieved papers (*statistical parameters published*), some more complete than others. However, in the majority of cases, our assessment showed that reconstructing a gender score from the provided parameters would be challenging.

We identified one paper that did not publish the gender score (*not published*) [31]. They adapted the scores from Nauman et al [11] and Pelletier et al [20] to form a new gender score, but they did not provide any specific information regarding their methodology.

Overall, repeatability was made difficult by many of the research groups, and the use of existing scores was made complicated. A systematic overview of the reproducibility of each study is given by our results in Multimedia Appendix 1.

#### Interrelations

We registered different dependencies and relationships between gender scores and visualized them (see Figure 2). Of the retrieved articles, 7 were developed with totally new approaches or methods (see Figure 2A).

Other scores adapted or included content-related parts of already existing scores (see Figure 2B). For example, Pelletier et al [20] included the BSRI masculinity and femininity scores [19]. Spence et al [32] were inspired by Rosenkrantz et al [41] for the development of the PAQ in 1975, and thereupon, Tibubos et al [30] developed a short form of the PAQ in 2022. It is notable that the BSRI [19] and PAQ [32] each form the basis of 2 other scores.

Sometimes research groups implemented the statistical or methodological approach of another team (see Figure 2C). For instance, the statistical approach of Pelletier et al [20] was frequently used as the basis for current gender scores, for example, by Wandschneider et al [9], Lacasse et al [10], Gisinger et al [35], Pohrt et al [29], Cipriani et al [36], and Yuan et al [39].

### Applicability on Clinical Routine Data

#### Evaluation Overview

We tested all retrieved scores and their published variables for whether they are available in the MII CDS. Initially, we planned to only test retrospective scores developed on clinical data. However, we found that this would limit the number of possible scores too much.

The evaluation of the applicability on clinical routine data required a careful and considerate approach, as variable names are often ambiguous and it is usually not possible to match them directly. This can, for instance, be seen in variables related to care work, which include “responsibility for caring for other people in the household” [29], “dinner is always prepared by someone else” [40], “caregiver strain” [17], “taking care of sick people” [38], “informal caregiving” [33], and “responsibility for caring for children” [34]. Even though some gender scores might be well described and could be used for other datasets, transferring the variables appropriately to a different dataset proved to be complex.

#### Applicability of Gender Scores on the MII CDS

In the comparison between the variables contained in gender scores and within the MII CDS, we used a two-step approach: We first tried to match the exact gender score variables against the MII CDS, and in case this remained unsuccessful, we extended the search to similar or generic terms.

We found that no variables of any gender score are covered in the MII CDS. This finding is surprising since a high number of variables covered in gender scores, such as occupation, level of education, civil status, children, household composition and size, and social support, are part of social history, which is routine clinical information.

Our principle finding regarding this question was that no gender score can be applied upon the MII CDS. One exception is the score from Teterina et al [37], which includes diagnostic codes and disease-specific data, which could be matched against the MII CDS. This score, however, is highly specific and not usable as a generalized approach. Currently, the MII CDS only encodes the administrative gender as male, female, undifferentiated, or diverse [43]. Administrative gender refers to the gender that is recorded or recognized in official documents (eg, the ID card). It may differ from the birth-assigned sex.

Overall, most scores were applied on population studies or included in studies from the outset. These gender scores frequently include personality traits and psychological assessments (eg, stress, loneliness scales), which are not part of routine clinical practice and, therefore, also not part of the MII CDS.

To summarize, there are no fitting variables for the MII CDS, and it is impossible to apply an existing gender score to the dataset in order to include gender-specific analysis on clinical routine data.

## Discussion

### Principal Findings

This work identified and reviewed state-of-the-art gender scores and gender identity measures. These were systematically assessed regarding their applicability and practicability in health research. Last, we tested their applicability on routine clinical data using the MII CDS as a reference dataset.

We found that no gender score is applicable on the MII CDS, because the variables required for the gender scores are not part of the CDS. However, many variables that are commonly included in gender scores are also assessed during a patient’s clinical stay. For instance, variables regarding personal background (eg, living with your partner or children, professional field, or self-employment) are often asked during a clinic stay to verify if specific therapies or treatments are feasible. Physicians need to know if patients have responsibilities at home, if they have support, and if they have a stable social environment. A person’s background is gender-dependent and influences not only clinical routine but also research data and therapy guidelines (eg, risk factors dependant on lifestyle). This is underlined by the information in Multimedia Appendix 2, which presents examples of current social history documentation practices in a German hospital compared against typical variables used in gender scores. Several variables related to gender scores can be directly inferred from these documentations; however, they are currently not included in the MII CDS. We therefore argue that the current lack of applicability of gender scores on clinical routine data results from missing structured data collection in or extraction methods from clinical information systems. Since the MII CDS is an ongoing effort to standardize research data from routine clinical practice, we recommend that the Person Module of the MII CDS should be updated and expanded to create a sufficient knowledge base for gender health research. Specifically, we formulated 4 action steps that will enable gender-specific analyses (see Figure 1): analyze social history data, collect social history data in a machine-readable form, integrate a 2-step gender identity measure during patient admission, and develop custom gender score approaches for clinical data.

### Recommended Action Steps

#### Analyze Social History Data

Social history is part of routine clinical data collected for many patients. However, the captured information is oftentimes not structured. It can be found in the doctor’s notes or additional study data if patients are included in clinical trials. As a first step toward evaluating what information is currently available, we suggest analyzing the full-text information in the patient records using natural language processing and large language models for data extraction. It will be interesting to learn what conclusions and predictions one could draw from the available information and how this information corresponds to the described gender scores. Typical social anamnesis texts are given in Multimedia Appendix 2, showcasing the low information content. A structured analysis of social history data is still ongoing, but the preliminary check of the information content already indicates that the practice of documentation must be significantly improved.

#### Collect Social History Data in a Machine-Readable Form

We recommend that key components of patients’ social histories — such as occupation, education, civil status, number of children, household composition and size, social support — be collected in a structured, machine-readable format. Instead of recording social history solely as narrative text in physician reports, clinical information systems should provide individual fields for each variable, allowing clinicians to select or enter information directly. This approach would reduce the effort required for data processing and aggregation, enable semantic annotation, and facilitate automated export and comparison across datasets.

Several national-level initiatives already exemplify such practices. Scandinavian countries, for instance, collect structured social and demographic data that can be integrated with health care data [44,45]. Similarly, the Canadian Institute for Health Information promotes the standardized collection of social and behavioral data across health care settings, including variables such as family composition, living arrangements, social support, and socioeconomic status. Existing research has also demonstrated how such information can be effectively captured using discrete, machine-readable fields rather than free-text formats [46].

A widely recognized standard that could be further adapted for this purpose is HL7 FHIR [47], which provides a structured framework for encoding social history elements in clinical IT systems. Future work should build on these existing resources — as well as our overview for gender scores — to develop a system that supports the structured capture of variables necessary for calculating gender scores. Although it is important to build future work upon existing standards, ideally, the analysis of clinical data items determining gender should be conducted before designing structured data capture forms for patient social history.

#### Integrate a 2-Step Gender Identity Measure During Patient Admission

Moreover, to include as many gender dimensions as possible, it would be beneficial to collect more gender-specific data. A promising approach would be to implement the 2-step gender identity measure [13] with open-ended answers during patient admission. Implementing a prospective approach, with minimal effort, would provide valuable insights into gender identity in everyday clinical practice. Although the DIVERGesTOOL [48] was originally developed as an extension of the 2-step approach for the German research context, it may also be well suited for use in patient admission settings. It adds a third question regarding whether differences in sex development have ever been medically diagnosed.

However, collecting such data must be approached with care, because asking gender-related questions at patient admission can involve sensitive issues and carries potential risks. For transgender, nonbinary, or gender-nonconforming individuals, disclosing gender identity may raise concerns about discrimination or biased treatment. Patients frequently withhold this information due to previous negative experiences and a lack of trust in health care providers [49,50]. Inadequate staff training and poorly designed forms can lead to misgendering or exclusion. To ensure respectful and safe care, it is essential to provide staff with proper training, use inclusive language, and implement supportive documentation systems.

#### Develop Custom Gender Score Approaches for Clinical Data

Researchers should think beyond the common gender score approaches and develop a gender score that is suitable to clinical data and not only population studies.

An innovative approach was carried out by Teterina et al [37], who used diagnostic codes of patients with traumatic brain injuries in Canadian emergency departments. Researchers should try similar approaches on general — nondisease-specific — clinical data with diagnostic or medication variables. Furthermore, developing a minimalistic retrospective score could prove advantageous in balancing prediction quality with the number of required variables, making the scores applicable even when limited information is available (eg, [40]).

Even though gender is gaining importance in health research, it is still far from being implemented. Our investigation shows that a gold standard is missing that accounts for variations across social groups (eg, including geographical regions, age, time). Due to the lack of a gold standard, some research groups use gender as a predictor for sex when developing gender scores, which contradicts the purpose of introducing gender in research — to move beyond biological sex and gather more detailed information. Solely theory-driven approaches, where experts determine the used variables and their scaling, cannot be validated.

Last, repeatability is a major challenge when aiming at implementing existing gender scores. Due to inconsistent research group practices, existing gender scores are often described only theoretically without providing detailed variables, questions, or complete information on variable weighting. We therefore urge authors to provide complete documentation and executable models when developing gender scores.

To achieve the necessary progress, every inclusion of gender into health research is beneficial, even if it might not be comprehensive or sufficient to cover all aspects.

### Limitations

Our collection of gender scores is based on a scoping review conducted to provide a broad overview of the topic. One limitation of this approach is that only one reviewer conducted the full-text screening and the categorization of the retrieved articles. However, during data extraction, more than 30% (7/22, 32%) of the publications were initially double-checked by a second domain expert [23]. The reviewers were in full agreement on all reviewed articles; thus, a single reviewer continued the data extraction. Moreover, our review does not claim to provide a complete list of all existing gender scores but does provide general statements on the current state of gender scores and their applicability in health research.

Moreover, the MII CDS might not be fully representative to test against population-based gender scores. Most gender scores were developed on population or specific patient data, while the MII CDS contains routine clinical data. However, it is important to include gender not only in population research but also in clinical research. This is why we selected the MII CDS as a target dataset for routine clinical data to test the practicability in this setting. As a result, we were able to show that gender scores are not yet a feasible instrument for routine clinical data and are able to make recommendations on how to update and extend the MII CDS in the future.

### Comparison With Prior Work

Ballering et al [26] highlighted that gender scores should be the minimum effort in epidemiological studies, but the community should go further than that because the retrospective application of a gender score implies a lack of gender consideration from the outset of the study (eg, the study design). Our findings found several shortcomings with currently available retrospective gender scores. Therefore, our results suggest that self-reported gender assessments are more precise and better suited to assess gender, confirming the theory of Ballering et al [26].

Moreover, we can confirm the findings of Ballering et al [26], specifically that the most common variables in gender scores are related to occupation, income, education, civil status, caregiving and household responsibilities, and ways of spending (leisure) time. Furthermore, our results showed that, even though several of these variables are collected in everyday clinic work, they are not processed and stored in a machine-readable way.

### Conclusions

Considering sex and gender enhances equity and research quality. Despite the high importance of the topic, it remains challenging to include gender into health research for several reasons. Ethical implications such as properly defining the concept of “gender” as opposed to “sex,” identifying appropriate ways of asking about a patient’s gender, and raising awareness of the importance with nurses and doctors are nontechnical reasons why the health gender gap remains. Technical implementation is hampered by the absence of a generally applicable clinical gender score. Structured, computer-processable data and metadata about gender-specific aspects in a patient’s social history is also lacking.

In this work, we identified, categorized, and systematically assessed state-of-the-art gender scores from epidemiology and clinical studies. We evaluated their applicability and practicability on a German national research dataset for routine clinical practice, the MII CDS. We found that gender cannot be predicted based on the MII CDS, even though several of the frequently used variables are part of routine clinical practice in Germany.

However, we see an urgent need to include gender-relevant information to the MII CDS in order to narrow the gap between routinely collected clinical data and available research data (see Multimedia Appendix 2). Therefore, further work is necessary to enable gender-specific analysis and to routinely collect more gender-specific data in clinics, for example, during patient admission.

Despite different approaches for assessing gender, no standardized and validated gender score that could be used retrospectively in clinical research exists.

Our study is limited to the German clinical landscape, and our evaluation of possible scores is based on a scoping review. Future investigations into the literature and common practices at clinics inside and outside Germany might give further insights and add to the action items suggested in this work.

## Supplementary material

10.2196/74162 Multimedia Appendix 1 Detailed information from the assessment of the retrieved gender scores.

10.2196/74162 Multimedia Appendix 2 Three patients showcasing the current social history documentation in clinical practice in a German hospital. Footnotes and color coding indicate the comparison with typical variables used in gender scores. The text examples have been constructed for illustration.

## Abbreviations

BSRI: Bem Sex Role Inventory
CDS: core dataset
MII: Medical Informatics Initiative
PAQ: Personal Attributes Questionnaire
