# Supplementary figures and images for: Applicability of Existing Gender Scores for German Clinical Research Data: Scoping Review and Data Mapping
Source: JMIR Med Inform. 2026 Jan 8;14:e74162. doi: 10.2196/74162 (PMC12782135; doi:10.2196/74162)

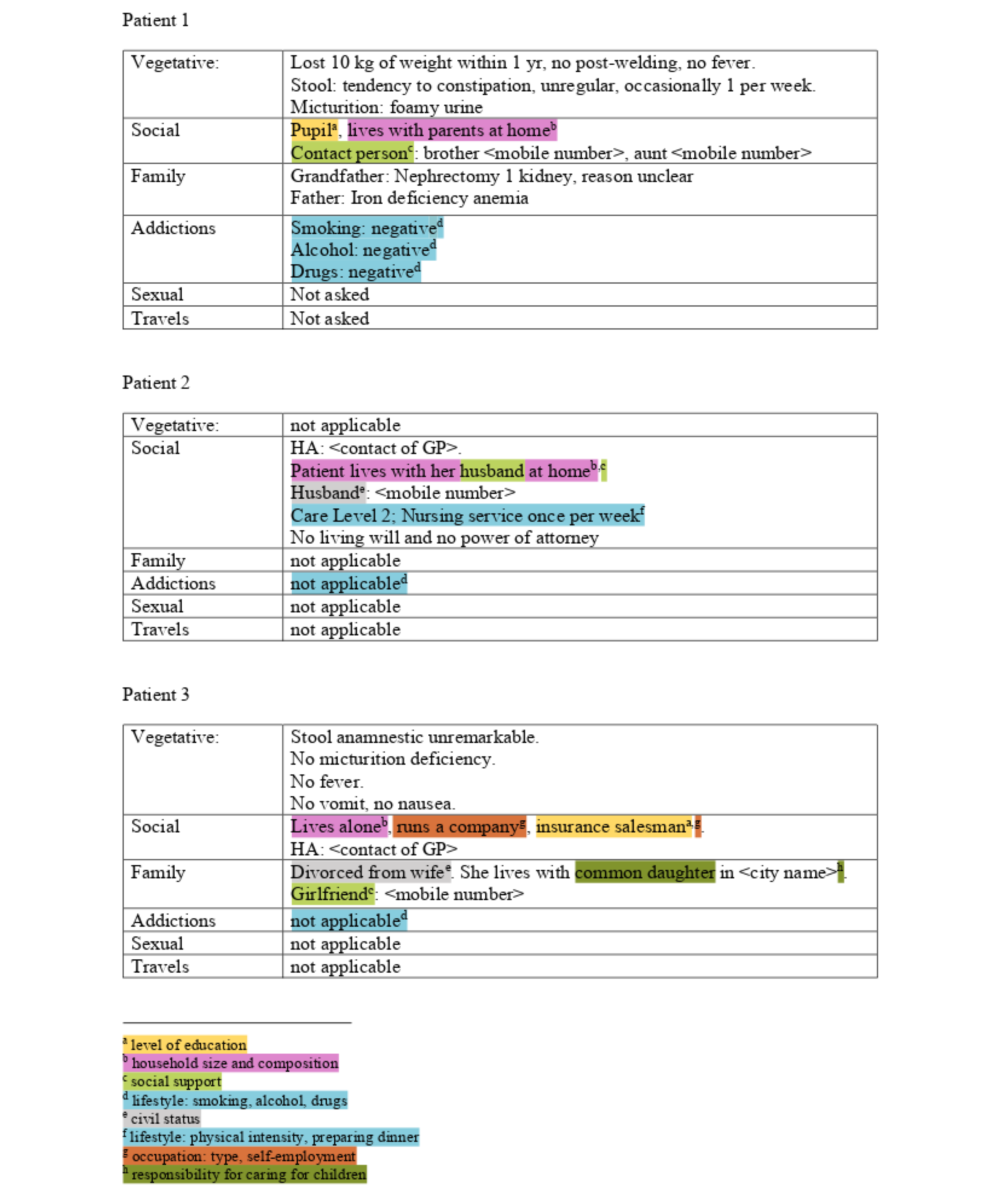

Supplement: Multimedia Appendix 2 [file medinform-v14-e74162-s002.png]
